# Neuroprotective effects of the *Chrysophyllum perpulchrum* extract against an Alzheimer-like rat model of β amyloid_1-40_ intrahippocampal injection

**DOI:** 10.1515/tnsci-2020-0183

**Published:** 2021-12-16

**Authors:** Pacôme Kouadio N’Go, Omar Touhami Ahmed Ahami, Aboubaker El Hessni, Fatima-Zahra Azzaoui, Youssef Aboussaleh, Antoine Némé Tako

**Affiliations:** Peleforo GON COULIBALY University, Training and Research Unit of Biological Sciences, Department of Animal Biology, PO Box 1328, Korhogo, Ivory Coast; Clinical and Cognitive Neurosciences Group, Biology and Health Lab, Ibn Tofail University, PO Box 133, Kenitra, Morocco; Genetic, Neuroendocrinology and Biotechnology Team, Biology and Health Lab, Department of Biology, Ibn Tofail University, PO Box 133, Kenitra, Morocco; Neurosciences Team, Biology and Health Lab, Department of Biosciences, Felix Houphouet Boigny University, 01 BPV 34 Abidjan 01, Abidjan, Ivory Coast

**Keywords:** Alzheimer’s disease, *Chrysophyllum perpulchrum*, recognition memory, spatial learning, microglia, oxidative stress markers

## Abstract

**Objective:**

Alzheimer’s disease (AD) is a threatening disease for African populations in the upcoming years because of the increase in their expectancy of life. Here, we investigated whether natural products from *Chrysophyllum perpulchrum* as catechin and two dimeric procyanidins (catechin + hexose) could prevent progression of oxidative stress and cognitive changes using an AD-like rat model induced by Aβ_1-40_ injection into the hippocampal CA1 subfield.

**Methodology:**

Adult male Wistar rats were either microinjected with 1% ammonia as a vehicle (10 µL) or aggregated Aβ_1-40_ at 10 µg bilateral hippocampus. On the 14th day of post-surgery, some Aβ rats were treated with melatonin (10 mg/kg i.p.) or with the *Chrysophyllum perpulchrum* extract (300 mg/kg p.o.), and some sham-operated rats received the extract alone. Cognitive abilities were tested with Y-maze, object recognition test and Morris Water Maze. Oxidative stress markers as well as the level of activated microglial cells were assayed in the brain.

**Results:**

Aβ rats exhibited significant deficits of recognition memory and spatial learning. This was associated with an increase of microglia Iba 1 immunoreactivity as well as nitric oxide (NO), malondialdehyde and superoxide dismutase levels but not to the thiol content in the hippocampus, prefrontal cortex and septum of AD-like rats. The *Chrysophyllum perpulchrum* extract treatment mitigated Aβ-induced cognitive impairments and reversed microglia overactivation and subsequent generation of oxidative stress markers. Interestingly, the neuroprotective actions of the *Chrysophyllum perpulchrum* extract seem to be comparable to the control drug melatonin used albeit with some more beneficial effects.

**Conclusion:**

These findings are preliminary and should be strengthened by more pharmacological studies of bioactive compounds of *Chrysophyllum perpulchrum* before being proposed as a promising drug against AD.

## 1 Introduction

Alzheimer’s disease (AD) is the most prevalent neurodegenerative disease with memory loss and cognitive decline in the elderly. From 47 million AD patients today, it is estimated to increase to about 130 million cases around 2050 [1], and approximately 7% of persons aged over 65 years and 40% of people over 80 years being afflicted in developed countries. However, populations of low-level income countries, including sub-Saharan Africa (SSA), could be more at risk in the upcoming years due to the improvement of the quality of lifestyle, or likely, the increase of life expectancy of populations.

Pathological hallmarks of AD are abnormal large quantities of β-amyloid (Aβ) deposition as senile plaques, intracellular neurofibrillary tangles of hyperphosphorylated Tau proteins and increase of cholinergic neurons degeneration [2,3]. Although all the factors triggering AD are not still clearly elucidated, it is reported that aggregated Aβ peptide plays a pivotal role in the pathogenesis. First, Aβ plaques originate from neuroinflammatory cascade events via microglia overactivation leading to irreversible neuronal death and AD severity [4,5]. Furthermore, a high affinity of Aβ towards redox-active metals as cooper or iron has been proven in several brain parts of AD patients [6]. Aβ has also the capacity to impair significantly the mitochondrial electron chain transport function [7]. All of these processes are major sources of reactive oxygen species (ROS) generation, exacerbating oxidative stress and neurotoxicity leading to neuronal death [8].

Otherwise, most approved drugs are until now derived from chemical synthesis, with high risks of side effects. In this context, new research studies focus on the beneficial effects of substances naturally occurring in medicinal plants against neurodegeneration-like AD. Authors reported that natural products with antioxidant potency could prevent and/or attenuate ROS actions [9]. Moreover, some traditional plants have been tested with success in clinical trials against AD [10,11], even if almost all the studies have been carried out on plants originating from South America or extreme Asiatic areas. Surprisingly, scarce research studies have valorized the pharmacological actions of medicinal plants from SSA countries for AD conditions. Among the promising natural products tested for their effects against AD; flavonoids-like quercetin, kaempferol, myricetin or glycetein demonstrated anti-amyloidogenic effects [12]. However, more studies are needed to extend the actions of natural molecules, mainly the ones from African medicinal plants in the AD context.

*Chrysophyllum perpulchrum* (Sapotaceae) is an endemic plant of tropical Africa’s forest found in Ivory Coast, Liberia, Cameroon and Uganda [13]. It is a potent antipyretic to heal malaria fever in Ivorian traditional pharmacopeia. *Chrysophyllum perpulchrum* contains three bioactive compounds including catechin (flavanol) and two dimeric procyanidins (tannins) with oxidative activity similar to quercetin after testing with DPPH [14]. Here, we promote neuroprotective actions of *Chrysophyllum perpulchrum* by using an *in vivo* model of AD. We aim to investigate whether the methanolic bark extract of *Chrysophyllum perpulchrum* could counteract neuroinflammation and oxidative stress processes in AD-like rats induced by intrahippocampal CA1 subfield microinjection of Aβ_1-40_ and relieve cognitive deficits.

## 2 Material and methods

### 2.1 Phytochemical compounds of the plant material

**Figure 1:**
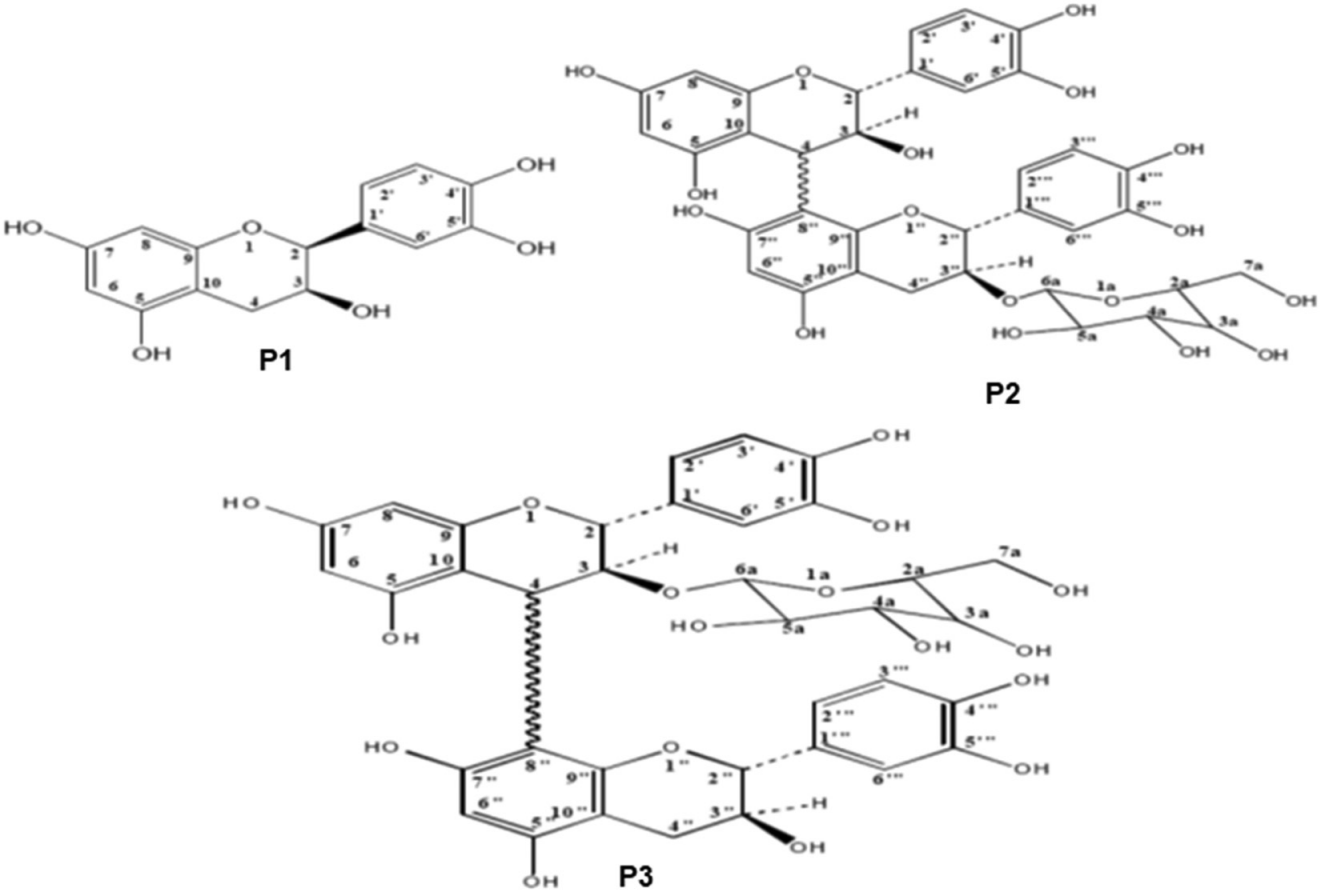
Chemical structures of compounds isolated from *Chrysophyllum perpulchrum* **P1**, catechin; **P2** and **P3**, two procyanidin dimers two dimeric procyanidins (catechin + hexose) [12].

The processes of methanolic extraction and total phenolic contents have been described elsewhere [14]. Bioactive compounds from *Chrysophyllum perpulchrum* as catechin (P1) and two dimeric procyanidins (P2 and P3) have been chromatographically characterized [14] (Figure 1). Acute toxicity study estimated the lethal dose 50 of *Chrysophyllum perpulchrum* to be 1,250 mg/kg of body weight (bw) according to the Organization for Economic Co-operation and Development procedure [14,15]. A next-door chemistry lab of Ibn Tofail University kindly gifted the plant extract.

### 2.2 Animals

Experiments were carried out on male Wistar rats (5- to 6-months old) provided by the local breeding house of Ibn Tofail University (Kenitra, Morocco). The animals were maintained for acclimatization under a controlled reference room at 22–25°C with good relative humidity, submitted to a 12 h light/12 h dark cycle and free access to standard food (ALF SAHEL Company of Casablanca) and tap water.


**Ethical approval:** The research related to animals’ use has complied with all the relevant national regulations and institutional policies for the care and use of animals. All experimental protocols were carried out according to the NIH guide for the care and use of laboratory animals and approved by the local Ibn Tofail university ethic committee.

### 2.3 Experimental design

The Aβ_1-40_ peptide (Sigma Aldrich, St. Louis, USA) was prepared as a stock solution in 1% ammonia at a concentration of 1 µg/µL, and aliquots were stored at −20°C. Before using, Aβ_1-40_ solution was aggregated by incubation at 37°C for 4 days, as described previously [16].

For the stereotaxic surgery, animals were submitted to general anaesthesia with chloral hydrate (400 mg/kg, i.p., in 7% solution) and were bilaterally injected into the hippocampus with a 10 µL Hamilton microsyringe (CA1 subfield according to the coordinates from Paxinos and Watson atlas 3.6 mm posterior from Bregma, ±1.8 mm lateral and 2.6 mm below the skull surface).

Animals were randomly divided into five experimental groups of 6–7 animals each: (1) the sham-operated group received 10 µL of 1% ammonia (as a vehicle); (2) the group of Aβ rats was microinjected with 10 µg of Aβ per side; (3) the group of Aβ + M rats, as a positive control, were microinjected with 10 µg per side and treated daily with 10 mg/kg bw i.p. of melatonin for 14 days; (4) the group of Aβ + CP rats were microinjected with 10 µg per side and treated with 300 mg/kg bw p.o. of methanolic bark crude extract *Chrysophyllum perpulchrum* for 21 days; and (5) the group of CP rats as sham-operated rats and treated with 300 mg/kg bw p.o. of the *Chrysophyllum perpulchrum* extract for 21 days.

All the treatments with either the extract or melatonin were carried out from 2 weeks post-surgery, a needed period for the Aβ to cause neuroinflammation and oxidative stress [17,18].

### 2.4 Cognitive testing

#### 2.4.1 Y-maze

This test was performed to evaluate the spatial working memory in rodents after all the treatments [19,20]. The apparatus was made of fine-wood with three arms (A, B and C) measuring 40 cm in length, 10 cm in width and 13 cm in height) and painted in different colour patterns. A central platform is formed by tri-angles of 120° between each arm. The procedure consists of giving 8 min-session to each rat for exploring freely the maze. The sequence of arms entries was monitored with a camera video. An entry is validated when the four paws are within the arms. Alternation is defined as a triad of successive entry in different arms (i.e. ABCACBAACB = 5 alternation). Spontaneous alternation behaviour was calculated with the following equation:

\% \hspace{.25em}\text{Alternation}=100\times (\text{Number}\hspace{.25em}\text{of}\hspace{.25em}\hspace{2em}\text{alternation/Total}\hspace{.25em}\text{arm}\hspace{.25em}\text{entries}-2).]

#### 2.4.2 Recognition memory test (NORT)

Novel object recognition testing is suitable to assess AD-related cognitive impairment [21]. The object recognition test procedure was conducted as described by Enanceur and Delacour [22]. The apparatus is an open box with a floor measuring 50 cm in length, 50 cm in width, and 40 cm in height walls. In the trial (familiarization session), rats were allowed 5 min to explore freely the box with two identical objects and return to their home cage. After 2 h delay, to evaluate short test recognition memory (STM), rats were returned to the open field in which one object was switched by another one different in colour, shape and size, and the observation was repeated for 5 min with one novel object and one previously explored one. To evaluate long-term memory (LTM) 24 h later from the familiarization phase, rats were submitted to explore again two objects for 5 min, one identical and another novel one. Objects and boxes were cleaned with 70% ethanol during the intertrial period. The exploration time of each object was recorded by video tracking, and the exploration is defined as the rat directing the nose at a distance less than 1 cm from the object. The ratio of preference of the novel object of each animal was calculated from the exploration frequency of the novel object divided by the total frequency spent for exploring both objects.

#### 2.4.3 Morris water maze (MWM)

The MWM was performed to evaluate spatial learning and reference memory. The apparatus was according to Morris et al. [23] with slight modifications. Thus, it consisted of a circular open light grey pool (120 cm in diameter and 40 cm in depth) filled with tepid water (22°C) at 30 cm of height. It is divided into four virtual quadrants (North, South, West, and East) each including a visual cue outside the surface of the water on the wall of the maze. In the habituation phase, animals were allowed 60 s to swim freely in the tank in order to define the best position of the platform that remained fixed during the all-test sessions. Then, in the training phase, rats were left in the tank facing the wall and then allowed to swim to find the hidden platform submerged to 1 cm to the surface of the water. If the animal did not find the platform after 60 s elapsed, it was gently guided to it and kept for 15 s before returning to the home cage. The testing was completed in five consecutive days including four trials each day. The behaviour of the rat was recorded by a video camera placed on the ceiling above the maze.

We considered the escape latency parameter as the time spent to find the platform.

### 2.5 Biochemical dosages

After behavioural testing, a part of each batch of rats was anesthetized with choral hydrate (400 mg/kg i.p.) and killed by decapitation. Brain regions corresponding to the hippocampal area, prefrontal cortex (PFC) and septal area were quickly removed and homogenized in ice-cold 50 mM Tris-HCl buffer (pH 7.4). The homogenate was then centrifuged at 3,000 rpm for 30 min at 4°C to obtain a supernatant stored at −20°C until assay.

#### 2.5.1 Nitrite oxide (NO) level assay

The quantification of NO content was determined by the nitrite level in a sample using the Griess reaction. Briefly, 100 µL of the supernatant was mixed with 50 µL of Griess reagent [(1% sulphanylamide (A) and 0.1% N-1-naphtylethylenediamine dihydrochloride (B) in 2.5% orthophosphoric acid)], and the mixture was incubated at 37°C for 10 min in the dark. The reaction was performed in two steps. The first one consisted of a dinitrogenation reaction between the nitrite and the Griess reagent A leading to a diazonium salt by-product. The second step is the formation of a stable chromophoric Azo product resulting from the coupling between the Griess reagent B and the diazonium salt. The Azo product strongly absorbs at 543 nm as read with an ELISA reader. Nitrite oxide concentration was expressed as µmol/L.

#### 2.5.2 Analysis of superoxide dismutase (SOD) level

The SOD activity was assessed in brain tissues according to the method described by Winterbourn et al. [24]. The principle is based on the ability of SOD to inhibit the reduction of nitroblue tetrazolium (NBT). The reagent medium was made with 1 mL of a solution containing 50 mM potassium phosphate buffer (pH 7.4), 1.5 mM methionine, 0.1 mM EDTA, 0.025% Triton X100, and 300 µL of the supernatant. The reaction was initiated by adding 0.12 mM riboflavin to each sample. After 10 min of incubation, the illumination of riboflavin in the presence of O_2_ and electron donor as methionine is a factor of generation of superoxide anions and SOD activity assay. The reduction of superoxide anion radical by NBT develops a specific colour that is monitored at 560 nm. The amount of enzyme needed to induce 50% inhibition of NBT reduction was termed 1 U of SOD, and expressed as U/mL of supernatant.

#### 2.5.3 Analysis of non-protein thiol (NP-SHs) level

The level of NPSHs was assessed according to the method described by Ellman [25]. The supernatant was treated with 10% of trichloroacetic acid to precipitate proteins, and the sample was centrifuged at 2.000 rpm for 10 min. To the supernatant, 1 M of potassium buffer (pH 7.4) and 1 mM Ellman’s reagent (5,5-dithiobis-2-nitrobenzoic acid) were added. The NPSH levels were determined at 412 nm and expressed as µmol/g of tissue.

#### 2.5.4 Determination of lipid peroxidation level

The malondialdehyde (MDA) assay as an index of the lipid peroxidation level was performed according to the method of Satoh [26]. Briefly, 500 µL of the supernatant corresponding to a hippocampus or CPF sample was mixed with 1.5 mL of trichloroacetic acid (10%), vortexed and incubated at room temperature for 10 min. Then, it was added to the mixture of 1.5 mL of thiobarbituric acid (0.67%), and heated in boiling water for 15 min. After cooling, 1.5 mL of n-butanol was added to the solution and thoroughly vortexed. The sample was centrifuged at 800 rpm for 5 min, and the supernatant was collected. The absorbance was determined spectrophotometrically at 532 nm. The results were expressed as MDA level µmol/g of tissue.

### 2.6 Immunohistochemistry (IHC)

After behavioural testing, rats were anaesthetized with chloral hydrate (400 mg/kg i.p.) and perfused through the posterior end of the left cardiac ventricle with 100 mL of PBS (0.1 M, pH 7.4) and then with 100 mL of a fixative solution (4% of paraformaldehyde in 0.1 M of sodium phosphate buffer, pH 7.4). The brain was carefully removed and kept overnight in the fixative solution at 4°C. On the following day, brain sections (50 µm) were done in 0.1 M phosphate buffer using a vibratome (VT 1000S, Leica microsystems). The IHC study was performed on free-floating brain sections corresponding to the CA1 subfield of the hippocampus and PFC. After several washes with PBS (0.1 M), the samples were treated with a quenching solution of endogenous peroxidase activity containing 1% hydrogen peroxide in 30% methanol under slight agitation at room temperature for 15 min. Then, the sections were washed 3 times in PBS and incubated with a solution of 10% normal goat serum, and 0.1% Triton X-100 in phosphate buffer (0.1 M, pH 7.4) for 30 min. The samples were immediately incubated with a previously diluted solution of 1% goat serum and 0.1% triton-X 100 in phosphate buffer for at least 5 min at room temperature. After these steps, the sections were incubated overnight in a cold room (+4°C) with a primary anti-Iba1 (monoclonal Iba 1; diluted at 1:1,500; Sigma Aldrich) as a marker of microglia activation. Sections were then washed 3 times and incubated for 2 h at room temperature with an anti-mouse biotin secondary antibody (1:200). After several washes, the sections were incubated with horseradish peroxidase-streptavidin (2 drops by tissue section, NeoSplink HRP broad spectrum kit with DAB) for at least 10 min at room temperature. The sections were rinsed with PBS and incubated with the developing product diaminobenzedine substrate (for high sensitivity, mix two drops of DAB chromogen substrate well with 1 mL of the DAB substrate buffer). The sections were then dehydrated, mounted onto gelatinized slides, coverslipped and observed under a Leica DMRB-E light microscope. The positively stained microglia cells (Iba 1 immunoreactive) were analysed and counted with Image J software.

### 2.7 Statistical analysis

The experimental data were expressed as mean ± S.E.M. (standard error of the mean). Data were analyzed using one-way ANOVA followed by Tukey *posthoc* test for multiple comparisons. *P* < 0.05 was considered statistically significant.

## 3 Results

### 3.1 Effects of the *Chrysophyllum perpulchrum* extract on cognitive abilities in intrahippocampal Aβ_1-40_-injected rats

#### 3.1.1 Spatial working memory in Y-maze

**Figure 2:**
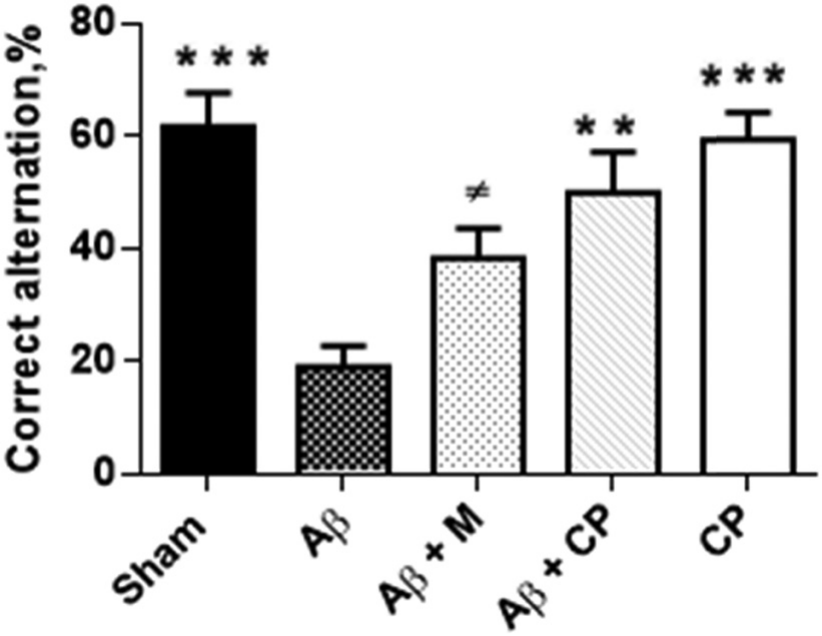
Effects of *Chrysophyllum perpulchrum* extract and melatonin treatments on working spatial memory in an AD-like rat model of hippocampal Aβ_1-40_ injection. Results are presented as the percentage of correct alternation; mean ± SEM (*n* = 6–7 animals, one-way ANOVA/*posthoc* Tukey test). **P < 0.01, ***P < 0.001 vs Aβ group; and ^±^ *P <* 0.05 vs Sham group.

The spontaneous alternation behaviour was used to evaluate the spatial working memory capacities. Data analysis revealed a significant difference in percentage of correct alternation between the studied groups (*F*_(4,27)_ = 10.08; *P* < 0.001). Aβ rats showed the lowest correct alternation score (19.11 ± 3.78%). However, the treatment of Aβ rats with 300 mg/kg of the *Chrysophyllum perpulchrum* extract significantly increased the percentage of correct alternation value (50.00 ± 7.18%, *P* < 0.01 vs Aβ rats), but not for the melatonin one (38.88 ± 5.27%, *P* > 0.05 vs Aβ group). The sham and sham-treated with plant extract groups performed better in the Y-maze compared to the Aβ group, either 61.92 ± 5.85 or 59.42 ± 4.71%, respectively (*P* < 0.001 vs Aβ rats) (Figure 2).

#### 3.1.2 Recognition memory in NORT

**Figure 3:**
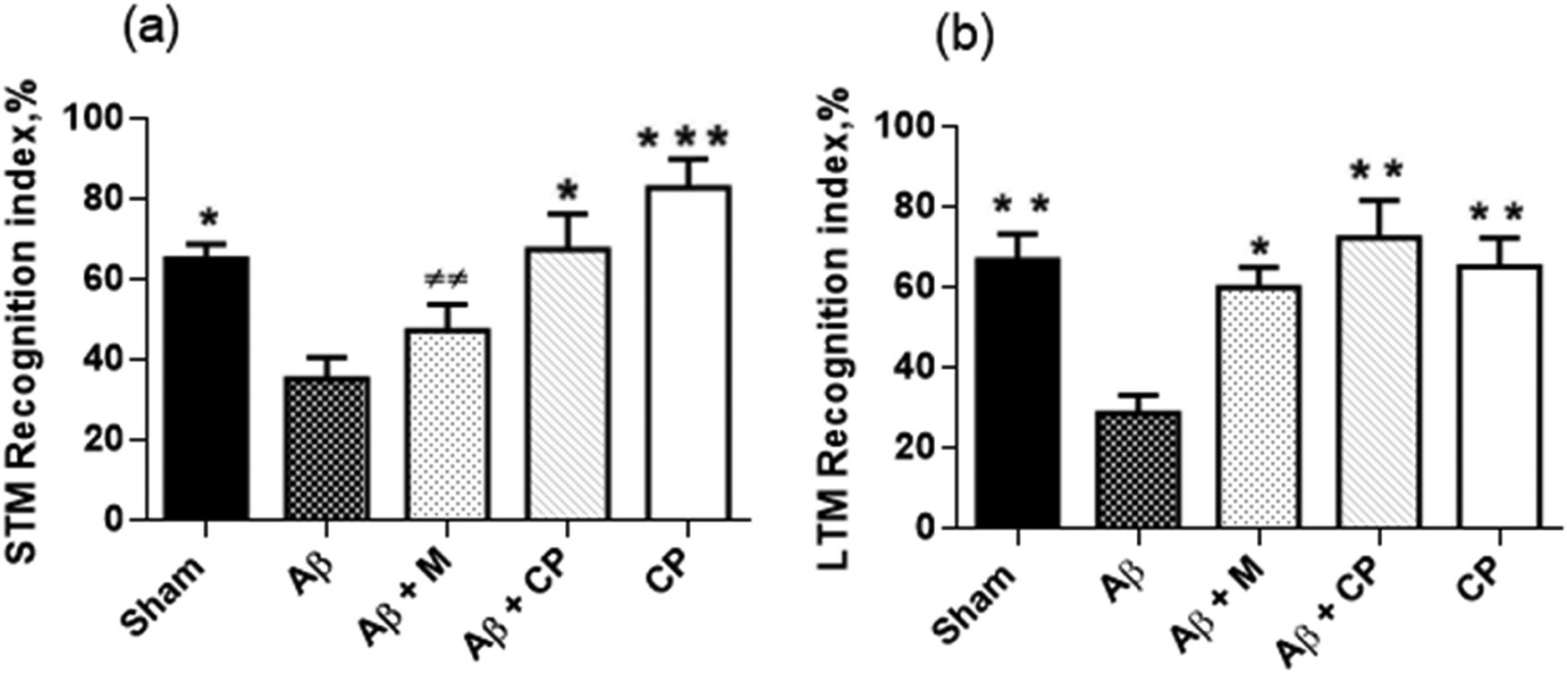
Effects of the *Chrysophyllum perpulchrum* extract and melatonin treatments on STM recognition (a) and LTM recognition (b) in an AD-like rat model of hippocampal Aβ_1-40_ injection. Results are presented as mean ± SEM (*n* = 6–7 animals, one-way ANOVA/*posthoc* Tukey test). *P < 0.05, **P < 0.01, ***P < 0.001 vs Aβ group, and ^±±^ *P <* 0.01 vs CP group.

The recognition index is based on the natural behavior to further interact with the novelty than the familiarity. The recognition index (%) during STM-recognition phase significantly varied between the studied groups (*F*_(4,27)_ = 8.33; *P* < 0.001). Aβ rats showed the lowest recognition index reported (35.43 ± 5.38%). The *Chrysophyllum perpulchrum* extract significantly protected Aβ_1-40_-induced STM-recognition impairment (67.78 ± 6.43%, *P* < 0.05 vs Aβ) compared to the treatment with melatonin (47.49 ± 6.43%, *P* > 0.05 vs Aβ rats) (Figure 3a).

The statistical analysis of LTM-recognition index showed also significant differences (*F*_(4,27)_ = 6.2; *P* < 0.01) (Figure 3b). Aβ rats lacked to interact more with novelty (28.75 ± 4.51%). However, all the treatments improved significantly LTM recognition deficit observed in Aβ rats; for Aβ rats-treated with the *Chrysophyllum perpulchrum* extract (72.50 ± 9.35%, *P* < 0.01 vs Aβ), Sham-operated group (66.90 ± 6.51%, *P* < 0.01 vs Aβ rats), Sham-operated-*Chrysophyllum perpulchrum-*treated group (65.30 ± 7.13%, *P* < 0.01, vs Aβ rats), and Aβ-treated with melatonin group (60.00 ± 5.00, *P* < 0.05 vs Aβ rats).

#### 3.1.3 Spatial learning and memory in MWM

**Figure 4:**
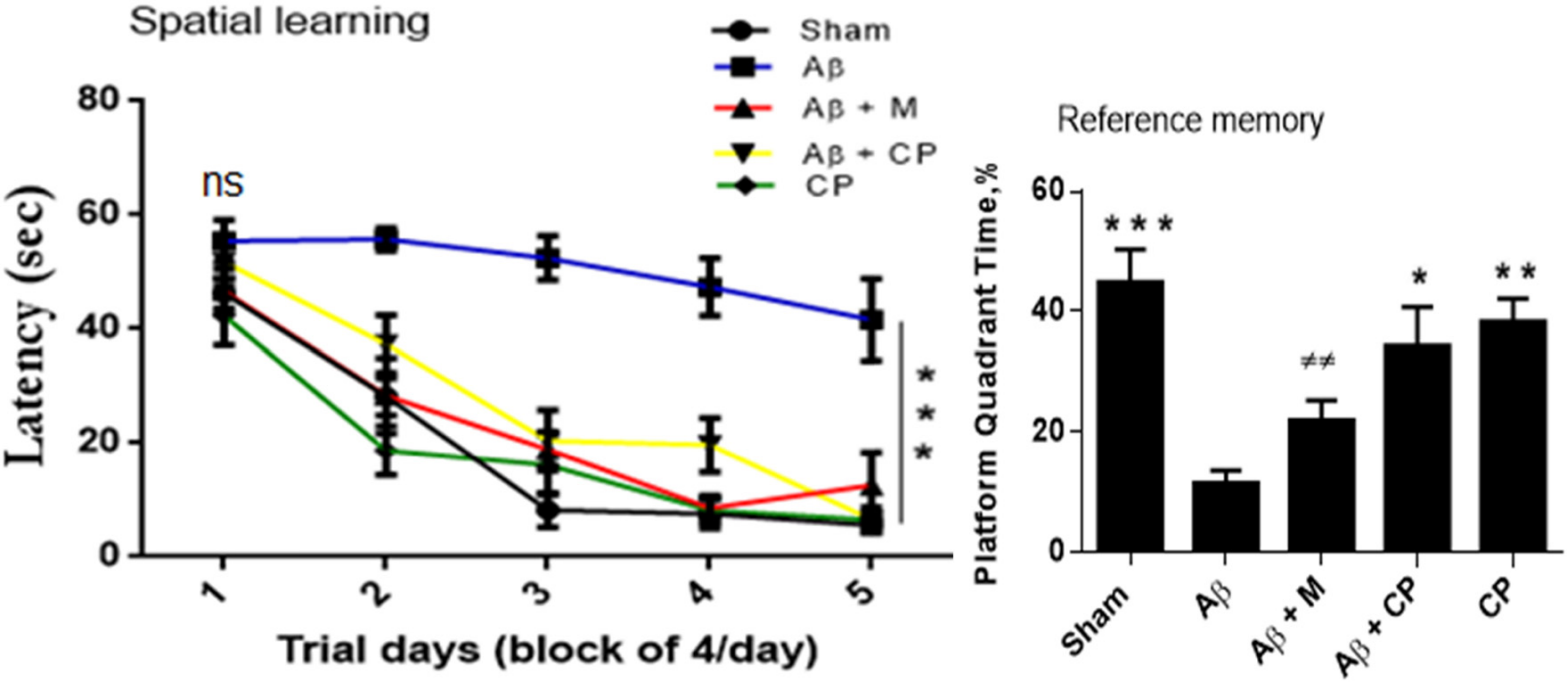
Effects of the *Chrysophyllum perpulchrum* extract and melatonin treatments on spatial learning and reference memory in an AD-like rat model of hippocampal Aβ_1-40_ injection. Results are presented as mean ± SEM (*n* = 6–7 animals, one-way ANOVA/*posthoc* Tukey test). *P < 0.05, **P < 0.01, ***P < 0.001 vs Aβ group, and ^±±^ *P* < 0.01 vs Sham group.

Our results showed that the treatment with the *Chrysophyllum perpulchrum* extract significantly reversed the effects of Aβ_1-40_ injection-induced learning and memory impairment in the MWM test, as well as the treatment with melatonin. In fact, on the 1st day of spatial acquisition, there were no significant differences in the escape latency time to find the hidden platform among the experimental groups. From the 2nd to 5th training days, we noted that Aβ rats exhibited poor spatial learning since no significant changes in the latency time to find the hidden platform were observed (from 55.45 ± 3.71 to 41.63 ± 7.20 s). In contrast, the Sham-operated rats learned faster to locate the platform (from 46.32 ± 2.67 to 5.56 ± 1.27 s, *P* < 0.001 vs Aβ rats), as well as Aβ rats-treated with plant extract (from 51.75 ± 2.79 to 6.59 ± 0.79 s, *P* < 0.001 vs Aβ rats), Aβ rats-treated with melatonin (from 46.65 ± 3.85 to 12.56 ± 5.79 s, *P* < 0.001 vs Aβ rats) and Sham operated-treated with plant extract (42.39 ± 5.17 to 6.52 ± 2.17 s, *P* < 0.001 vs Aβ rats; Figure 4).

Besides, we evaluated the reference memory ability by the time spent in the virtual platform quadrant area. During the 60 s of probe trial session, Aβ rats spent the lowest time in the platform quadrant area (11.55 ± 2.1% s) comparable to Aβ rats treated with melatonin (21.99 ± 3.31%, *P* > 0.05 vs Aβ rats), but incomparable to that of Aβ rats treated with the *Chrysophyllum perpulchrum* extract (34.27 ± 6.44%, *P* < 0.05; Figure 4).

### 3.2 Effects of the *Chrysophyllum perpulchrum* extract on the intrahippocampal Aβ-induced oxidative stress status

#### 3.2.1 Analysis of the NO level in brain areas

**Figure 5:**
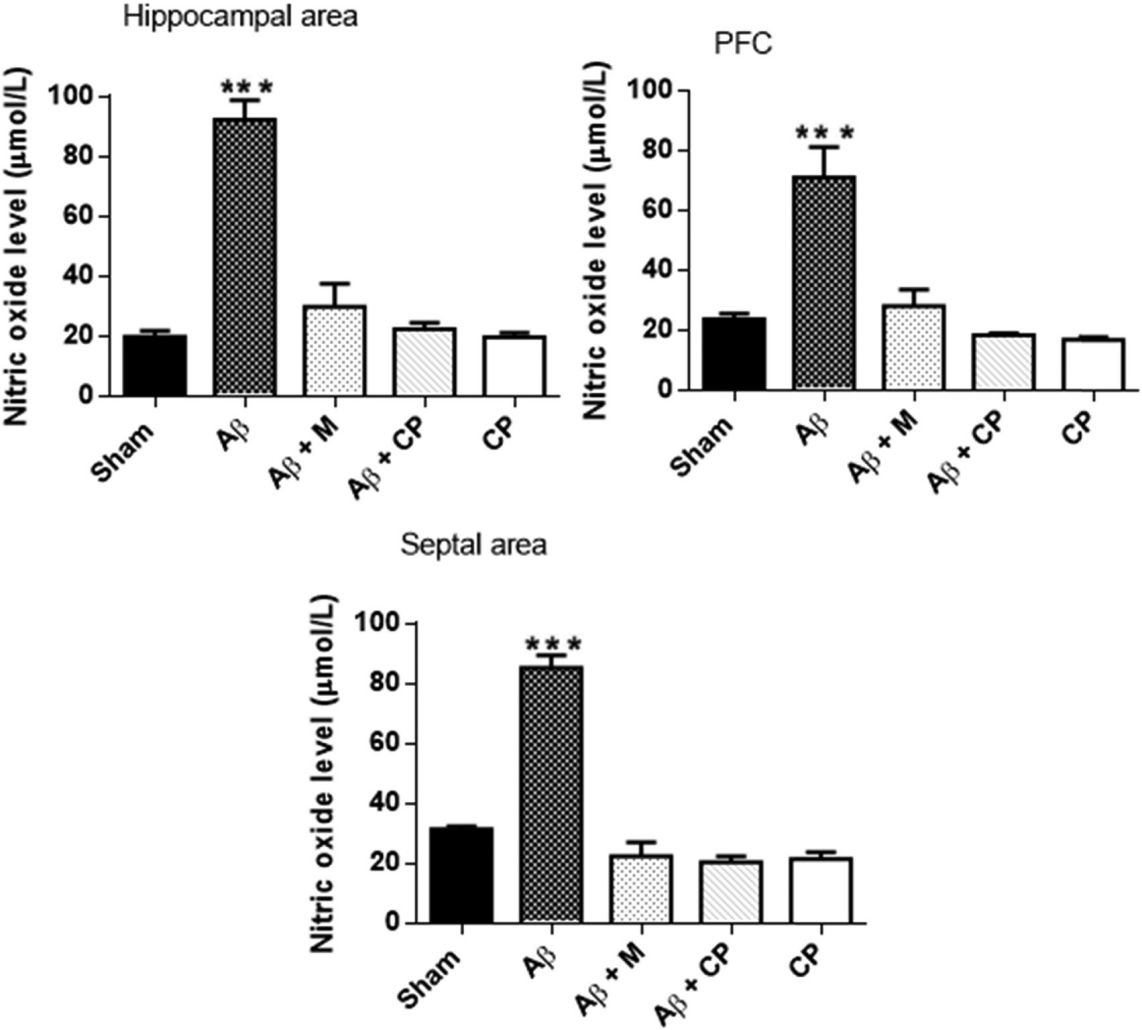
Effects of *Chrysophyllum perpulchrum* extract and melatonin treatments on NO level in an AD-like rat model of hippocampal Aβ_1-40_ injection. Results are presented as mean ± SEM (*n* = 6–7 animals, one-way ANOVA/*post hoc* Tukey test). ***P < 0.001 vs Aβ group.

The NO release is a subsequent effect of Aβ-induced neurotoxic microglial overactivation. In Aβ rats, there was a highly significant level of NO in all the brain parts studied; hippocampus (92.30 ± 6.41 µmol/L, *P* < 0.001), PFC (71.09 ± 10.23 µmol/L, *P* < 0.001), septum (85.20 ± 4.13 µmol/L, *P* < 0.001), when compared with other experimental groups. The treatment with *Chrysophyllum perpulchrum* extract attenuated at the best the NO level than that of melatonin without significant differences (*P* > 0.05) as the following: hippocampus (22.29 ± 2.9 vs 29.63 ± 7.75 µmol/L), PFC (18.36 ± 0.36 vs 28.03 ± 5.6 µmol/L), septum (20.26 ± 2.12 vs 22.33 ± 4.63 µmol/L) (Figure 5).

#### 3.2.2 Analysis of the SOD level in brain areas

**Figure 6:**
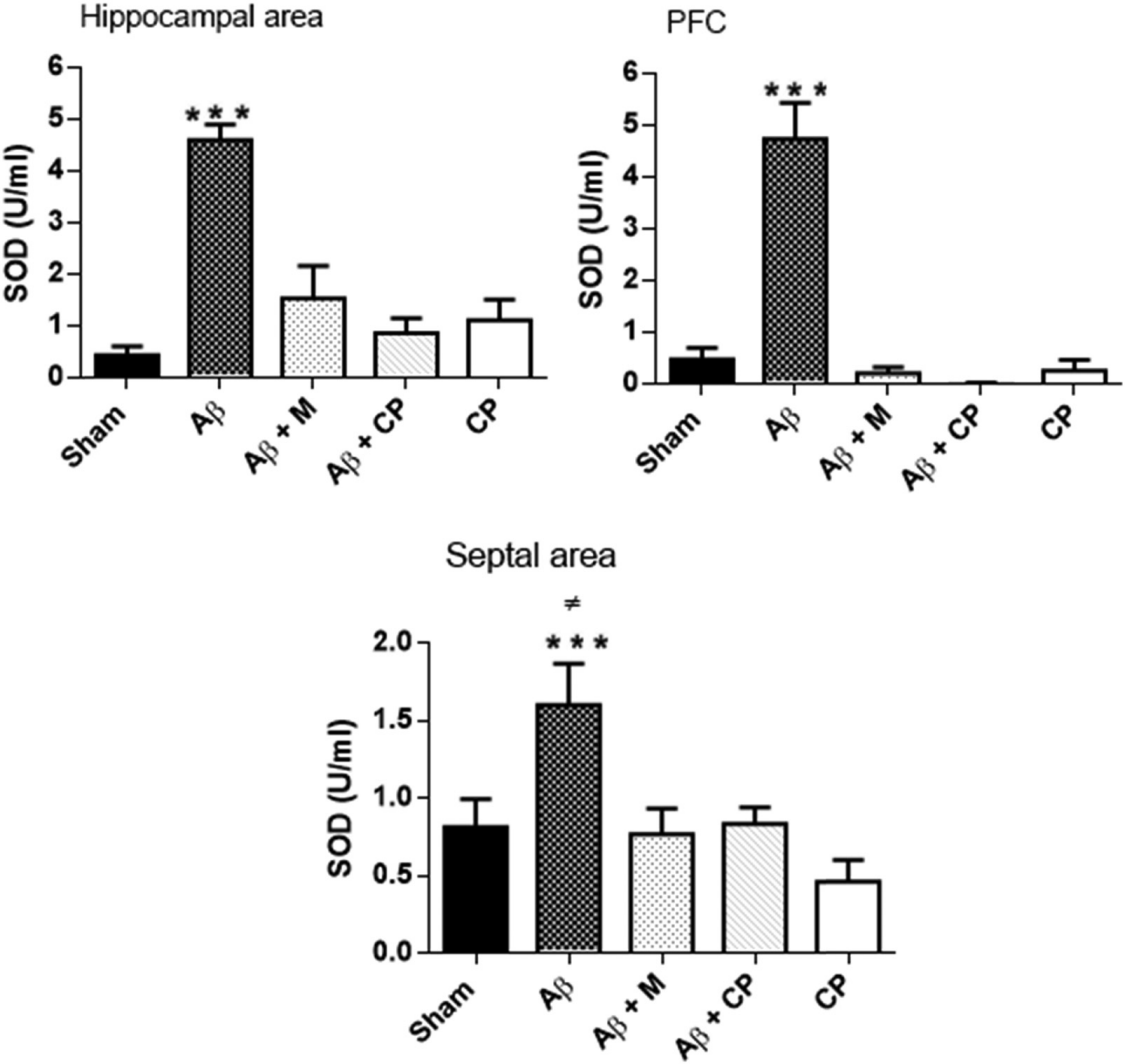
Effects of the *Chrysophyllum perpulchrum* extract and melatonin treatments on SOD activity in an AD-like rat model of hippocampal Aβ_1-40_ injection. Results are presented as mean ± SEM (*n* = 6–7 animals, one-way ANOVA/*post hoc* Tukey test). ***P < 0.001 vs Aβ group; ^±^ *P* < 0.05 Aβ group vs Sham, Aβ + M, and Aβ + CP.

The SOD activity in brain areas is represented in Figure 6. The activity of SOD increased more than 4-fold in the hippocampus (4.6 ± 0.29 U/mL, *P* < 0.001) and the PFC (4.73 ± 0.9 U/mL, *P* < 0.001) of Aβ rats, but decreased in the septal area (1.60 ± 0.29 U/mL, *P* < 0.05). The treatment of Aβ rats with 300 mg/kg the *Chrysophyllum perpulchrum* extract reversed significantly the SOD activity in the hippocampus (0.86 ± 0.28 U/mL) and in the PFC (0.013 ± 0.008 U/mL), but less much in those treated with melatonin either (1.63 ± 0.53 U/mL) in the hippocampus or (0.20 ± 0.12 U/mL) in the PFC. On the other hand, in the septal area, no significant changes in the SOD activity were found in both Aβ rats treated with the *Chrysophyllum perpulchrum* extract and with melatonin (Figure 6).

#### 3.2.3 Analysis of the NP-SH level in brain areas

Statistical analysis revealed highly significant differences in the NP-SH level in brain areas, in the hippocampus (*P* < 0.001) and in the PFC (*P* < 0.001), but less significant in the septum (*P* < 0.05) between the studied groups.

**Figure 7:**
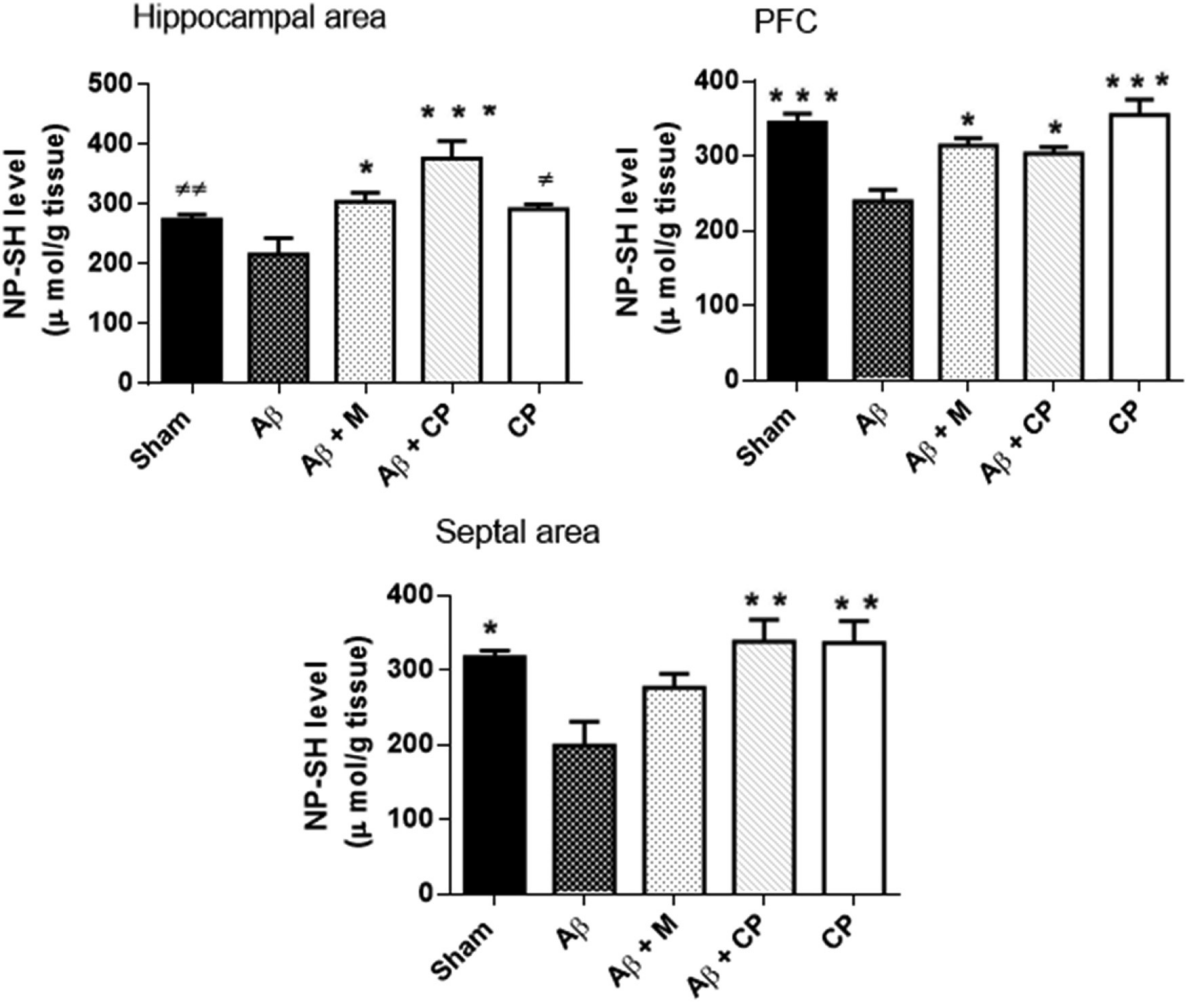
Effects of the *Chrysophyllum perpulchrum* extract and melatonin treatments on the NP-SH level in an AD-like rat model of hippocampal Aβ_1-40_ injection. Results are presented as mean ± SEM (*n* = 6–7 animals, one-way ANOVA/*post hoc* Tukey test) *P < 0.05, **P < 0.01, ***P < 0.001 vs Aβ group, ^±^ *P* < 0.05, ^±±^ *P* < 0.01 vs Aβ + CP.

*Post hoc* comparison showed that in hippocampus, Aβ_1-40_ injection significantly decrease the level of NP-SH (215.02 ± 27.74 µmol/g), but the *Chrysophyllum perpulchrum* extract increased the level of NP-SH (376.43 ± 28.74 µmol/g, *P* < 0.001 vs Aβ group), and also for melatonin treatment (303. 81 ± 14.77 µmol/g, *P* < 0.05 vs Aβ group). Similarly, in the PFC, we noted a decrease of the NP-SH concentration in Aβ rats (239.89 ± 15.16 µmol/g). It was enhanced by the treatment with the plant extract (314.41 ± 10.21 µmol/g, *P* < 0.05 vs Aβ rats) as well as with the melatonin treatment (303.9 ± 8.74 µmol/g, *P* < 0.05 vs Aβ rats). Finally, in the septum, the *Chrysophyllum perpulchrum* extract prevented an Aβ-induced significant decrease of the NP-SH concentration (*P* < 0.01) but not for melatonin treatment (*P* > 0.05) (Figure 7).

#### 3.2.4 Analysis of the lipid peroxidation level in brain areas

The MDA level differently varied in the brain areas between the experimental groups, in the hippocampus (*P* < 0.001), in PFC (*P* < 0.05) and in the septal area (*P* < 0.001).

**Figure 8:**
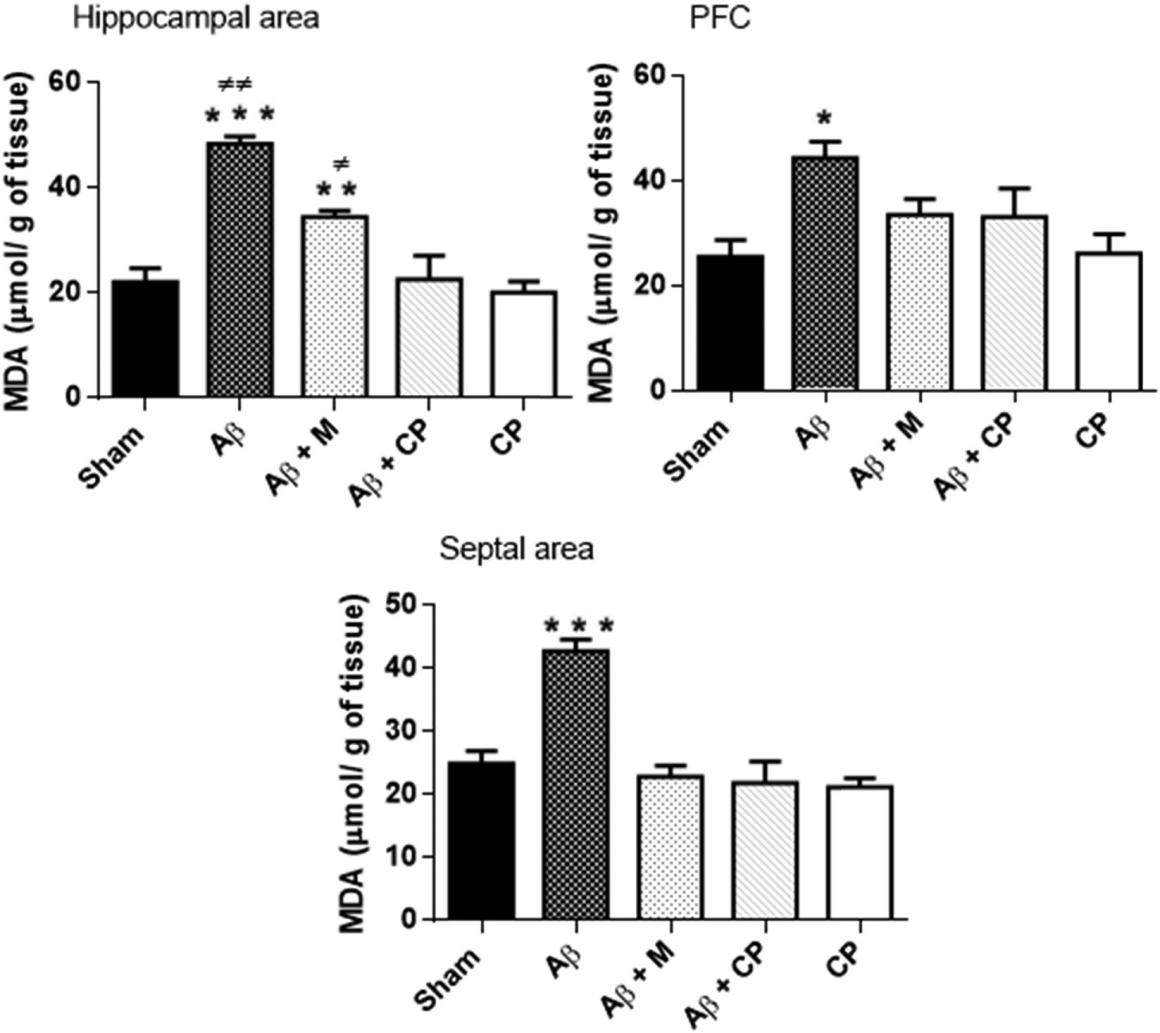
Effects of the *Chrysophyllum perpulchrum* extract and melatonin treatments on MDA levels in an AD-like rat model of hippocampal Aβ_1-40_ injection. Data are presented as mean ± SEM (*n* = 6–7 animals, one-way ANOVA/*post hoc* Tukey test). *P < 0.05, ***P < 0.001 vs Aβ group, **P < 0.01 vs CP; ^±^ *P* < 0.05 vs Sham or Aβ + CP, ^±±^ *P* < 0.01 vs Aβ + M.

*Post hoc* analysis revealed that in the hippocampus, Aβ induced a significant increase of the MDA level (48.96 ± 1.39 µmol/g of tissue), but the treatment with melatonin significantly scavenged the MDA level (34.50 ± 1.62 µmol/g of tissue, *P* < 0.01 vs Aβ group), and more significantly with the treatment of the *Chrysophyllum perpulchrum* extract (22.63 ± 4.54 µmol/g of tissue, *P* < 0.001 vs Aβ group). In the CPF, although Aβ injection increased significantly the level of MDA (*P* < 0.05 vs other groups), no difference was observed between melatonin and plant extract treatments. In the septum, both melatonin and the *Chrysophyllum perpulchrum* extract treatments reversed in the same manner the MDA level observed in the Aβ group (*P* < 0.001 vs other groups) (Figure 8).

### 3.3 Effects of the *Chrysophyllum perpulchrum* extract on Aβ-induced microglial cells’ activation

**Figure 9:**
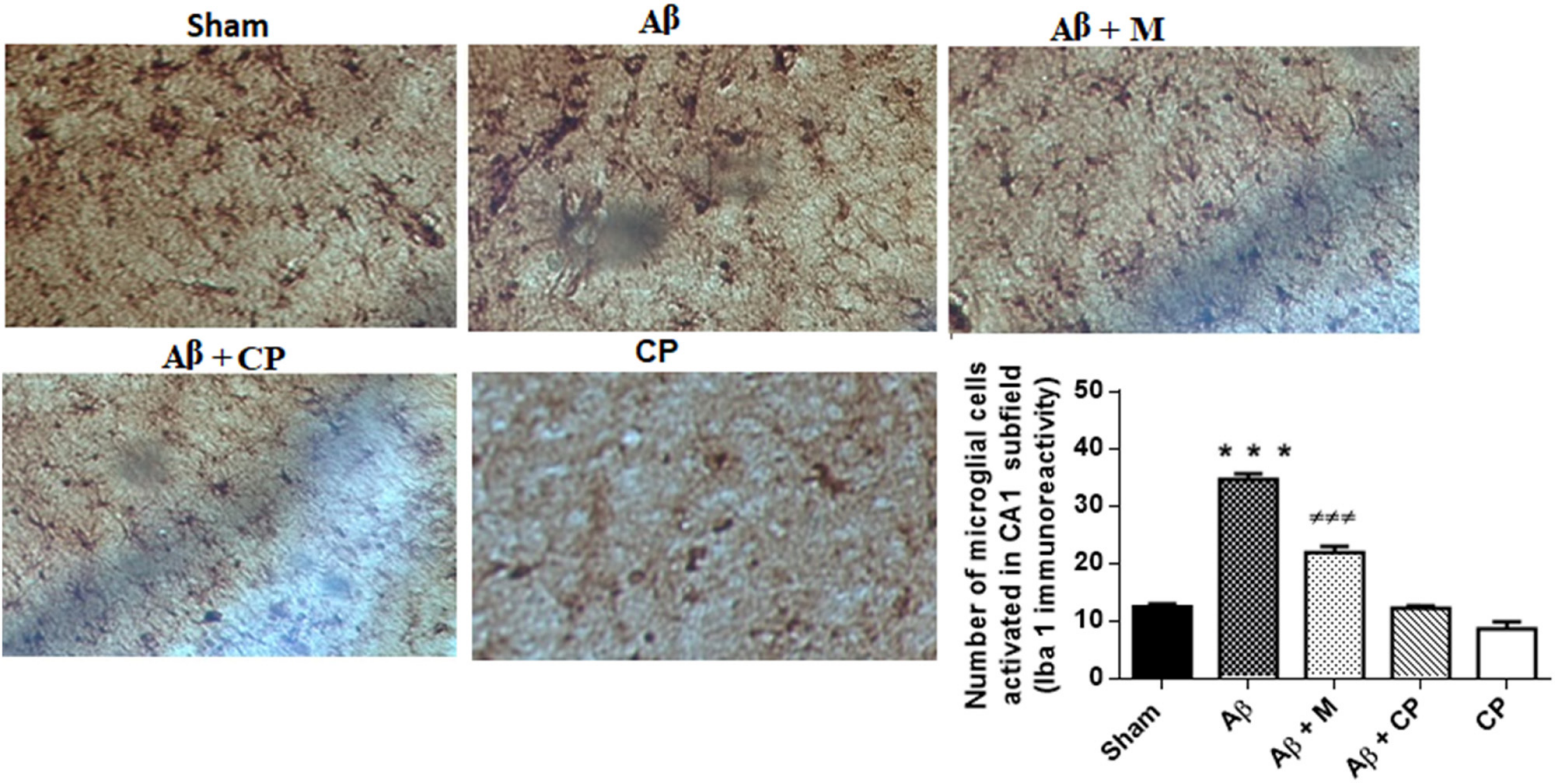
Effects of the *Chrysophyllum perpulchrum* extract and melatonin treatments on microglial cells’ activation in the hippocampal CA1 subfield area in an AD-like rat model of hippocampal Aβ_1-40_ injection. Data are presented as mean ± SEM (*n* = 2–3 animals, one-way ANOVA/*post hoc* Tukey test). ^***^ *P* < 0.001 vs Aβ, ^±±±^ *P* < 0.001 vs Aβ + M. Magnification: ×40.

Figure 9 indicates a fraction of the hippocampal CA1 subfield of immunoreactivity for Iba 1. The number of microglial cells activated was significant in Aβ rats when compared to other experimental groups (*P* < 0.001). The treatment with the *Chrysophyllum perpulchrum* extract attenuated the Aβ-induced microglia activation compared to the melatonin (*P* < 0.001).

**Figure 10:**
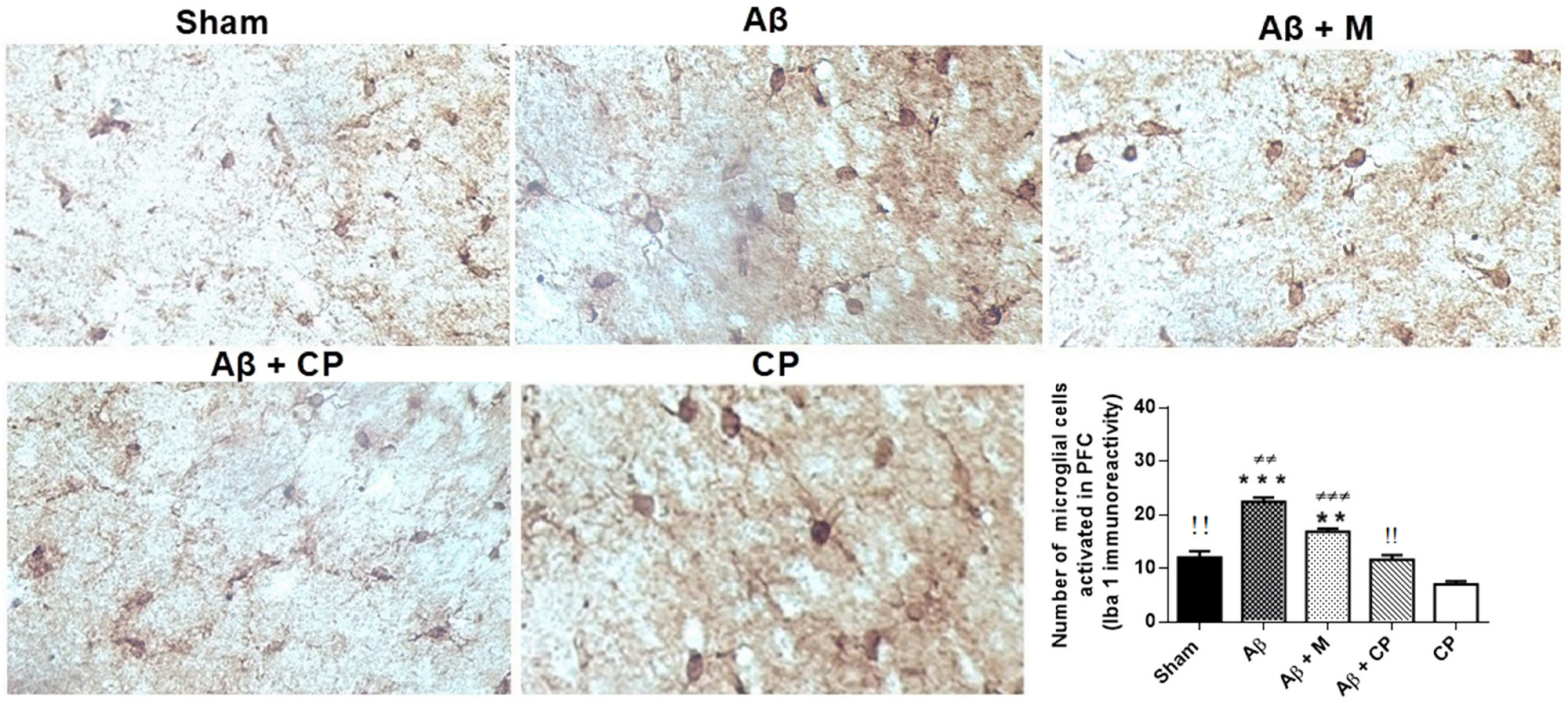
Effects of the *Chrysophyllum perpulchrum* extract and melatonin treatments on microglial cells’ activation in PFC in an AD-like rat model of hippocampal Aβ_1-40_ injection. Data are presented as mean ± SEM (*n* = 2–3 animals, one-way ANOVA/*post hoc* Tukey test). ^**^ *P* < 0.01 vs sham or Aβ + CP, ^***^ *P* < 0.001 vs sham, Aβ + CP or CP; ^±±^ *P* < 0.01 vs Aβ + M, ^±±±^ *P* < 0.001 vs CP, ^!!^ *P* < 0.01 vs CP. Magnification: ×40.

In the PFC area, the number of microglia activated increased approximately 2-fold in the Aβ group of rats as compared to other experimental groups (*P* < 0.001, Aβ group vs other experimental groups). However, the treatment of Aβ rats with *Chrysophyllum perpulchrum* reduced significantly the activated microglia compared to melatonin treatment (*P* < 0.01) (Figure 10).

## 4 Discussion

It has been reported that neuroinflammation and oxidative stress remain two consecutive mechanisms contributing to AD pathogenesis. Therefore, finding new multifunctional drugs targeting these processes might be a proper strategy to prevent cognitive disorders and neuronal death related to AD. Here, we tested whether the treatment with *Chrysophyllum perpulchrum* bark extract could have neuroprotective effects against AD-like changes in rat’s model with intrahippocampal Aβ_1-40_ injection. In our results, we found that Aβ rats exhibited significant deficits of the object recognition memory, spatial working memory and spatial learning capacities after testing. These cognitive changes in Aβ rats are accompanied by an increase of microglial cells in the hippocampal CA1 subfield and CPF areas, an increase in the levels of NO and SOD activity, as well as lipid membrane peroxidation. According to previous reports, a wealth of information is linking an exacerbation of Aβ-activated microglia via TLR (toll-like receptor) and upregulated NF-kB signalling for release of the cytokines (TNF-α and IL-1β), which in turn contribute to facilitate AD pathogenesis associated with a subsequent neuronal death [5,27]. Simultaneously, Aβ acts on the NF-kB pathway leading to overexpression of inducible-nitric oxide synthase (i-NOS) for the production of NO [28] and promotes ROS generation through the NADPH oxidase system [29]. These processes are occurring at first in microglia and expand towards the astrocytes before causing the loss of the cholinergic neurons. The basal forebrain nuclei predominantly the medial septum is the greatest provider of cholinergic neurons projecting on the hippocampus for memory formation modulation. In AD conditions, Aβ plaques are found early in the septum area causing its degeneration leading to memory loss and cognitive disorders [30,31]. Furthermore, a previous study of rat’s model using an Aβ_1-40_ hippocampal CA1 subfield injection damaged the septo-hippocampal axon terminals and favour their degeneration [32]. Our results on oxidative stress biomarkers showed a high level of the free radical NO generated in the septum area of Aβ rats, suggesting the degree of oxidative stress supporting poor memory and cognitive performances. Besides, Aβ-facilitated ROS generation such as hydrogen peroxide (H_2_O_2_) and hydroxyl radical (OH.) via copper or iron interaction [33–35], which in turn are potent molecules leading to lipid membrane peroxidation or DNA damage. In our experiment, we did not assess the pro-oxidant ROS levels. However, our results demonstrated an excessive SOD activity (antioxidant enzyme for ROS) in the hippocampus, CPF and septum areas of AD-like rats when compared to the other groups. That could be explained by some compensatory mechanisms to overcome the ROS burden. Our results are in agreement with others showing that Aβ injection (10 µg) significantly increased SOD level in the hippocampus and frontal cortex of a mice model of AD [36]. The increase of antioxidant enzymes in the hippocampus and amygdala of AD patients reported elsewhere corroborate with the observations in our AD-like rat model [37]. In our study, a possible increase of the ROS activity is also assumed by the high level of lipid peroxidation through high concentrations of its biomarker MDA in the different brain areas of the Aβ group of rats studied. In fact, ROS-mediated lipid peroxidation is an index of irreversible cell membrane damage leading to MDA formation. A former study reported interesting correlations between ROS, MDA levels and recognition memory impairment when studying an AD rat model by Aβ intrahippocampal injection [38].

Further effects of Aβ-induced neurotoxicity are closed due to neuroinflammation and oxidative stress conditions in AD [8,39]; thus, a possible therapy approach could be based on individual or multifunctional drugs for quenching toxicity markers, preventing neurodegeneration and ultimately cognitive disorders. To this end, our treatment proposal of AD-like rats with the *Chrysophyllum perpulchrum* extract has restored recognition memory and spatial learning deficits but also reduced oxidative stress status in the brain. The Aβ rats co-treated with the plant extract showed significantly lower levels of MDA, assuming to be able to counteract neuronal membrane peroxidation. Moreover, the treatment of AD-like rats with the *Chrysophyllum perpulchrum* extract demonstrated an attenuated activity of SOD in the brain regions mainly including the hippocampus. This suggests that the extract contains some chemicals enabling to co-manage with SOD by efficiently scavenging the ROS in order to prevent oxidative stress-induced memory impairment and neuronal death. This means otherwise that *Chrysophyllum perpulchrum* has some potential neuroprotective properties against Aβ-induced hippocampal injury, which is the brain part sensitive to oxidative stress [40]. Interestingly, we found an approximately 3-fold decrease of NO level in different brain areas after treatment of Aβ rats with the *Chrysophyllum perpulchrum* extract, suggesting its repressive action on the Aβ-induced iNOS activity as well as the release of the cytokine via downregulation of NF-kB expression. In the present work, we found also that Aβ rats treated with the *Chrysophyllum perpulchrum* extract showed a significant reduction of activated microglia in hippocampal and CPF areas. This is supposed to be accompanied by a low level of pro-inflammatory cytokines responses.

As mentioned above, by high-performance chromatographic method, Bidié et al. characterized two bioactive molecules of flavonoid from the *Chrysophyllum perpulchrum* extract; catechin and two dimeric procyanidins [14]. Schimidt et al. have reported a supposed neuroprotective action of catechin on Alzheimer’s-like disease [38]. They found that supplementation with green tea rich in catechin alleviated memory deficits and oxidative stress in the hippocampus of AD rats caused by an intrahippocampal Aβ injection. Another experiment reinforced that green tea catechin targeted abnormal accumulation of Aβ aggregation, oxidative stress, and elevated expression of pro-apoptotic proteins leading to cerebral cortex neurons dysfunction and death [41]. The catechin or its stereoisomer compound epigallocatechin gallate (EGCG) antagonizes Aβ-induced apoptosis by regulating some neuronal signalling pathways. For instance, some previous research studies reported that catechin or EGCG treatment caused an upregulation of the mitochondrial Bcl2 factor, which in turn downregulated pro-apoptotic proteins expression as Bax and caspase 3 in an experimental model of AD [36,42]. In addition to its anti-neuroinflammation, antioxidant or anti-apoptosis activity, catechin appears to be an important anti Aβ-aggregation compound. In fact, catechin sourced from the folk medicine plant *Rhizophora mucronata* has been tested as an inhibitor of β-secretase 1 and AChE reducing so Aβ peptide level [36], and that illustrates further the plausible beneficial actions attributed to catechin from *Chrysophyllum perpulchrum* used in our AD model. Moreover, EGCG from green tea reduced the Aβ level in the brain by the modulation of amyloid peptide precursor processing [43] and prevented Aβ aggregation by stimulating α-secretase or disrupting unfolded peptides [44].

A supplementary reason to explain the full neuroprotective effects of *Chrysophyllum perpulchrum* against AD might be based on its two dimeric procyanidins. In this sense, Kanno et al. revealed, for instance, that procyanidin B1 inhibitory activity on caspase 9 and 8 helped to prevent oligomeric Aβ-induced neuronal death [45]. Also, procyanidin A2 was used to suppress Aβ-induced neuroinflammation or apoptosis in the BV2 microglia cell line by upregulating Bcl-2 and downregulating Bax proteins’ expression [46].

The positive effects of the *Chrysophyllum perpulchrum* extract were compared to that of melatonin, which is known as a promising drug for AD therapy [47]. These authors reported abundant pieces of evidence of neuroprotective effects of melatonin including anti-inflammatory action, anti-amyloidogenesis, free radical scavenging activity, and prevention of Aβ-induced depletion of Bcl-2. Likewise, melatonin (10 mg/kg i.p.) administration for 14 days significantly improved spatial working memory in the intrahippocampal Aβ injection-induced rat model of AD [48]. In our study, we showed that the treatment of AD-like rats with *Chrysophyllum perpulchrum* was sometimes more efficient compared to melatonin. This is well shown with cognitive tests for recognition and reference memories as well as spatial working memory. The *Chrysophyllum perpulchrum* extract attenuated the activation of microglial cells and membrane lipid peroxidation at the site of Aβ injection (i.e. hippocampus CA1 sub-field) compared to melatonin. This would imply that the bioactive compounds of *Chrysophyllum perpulchrum*, catechin and new procyanidins (dimer of catechin + hexose), present a greater therapy effect against AD than melatonin.

A noticeable discrepancy noted was with the antioxidant SOD activity that was increased in Aβ rats and diminished in Aβ rats treated with plant extract or melatonin. The previous research mentioned a significant decrease of SOD activity due to a low amount of Aβ_1-40_ (2 nmol), bilaterally injected in hippocampus, but genistein (an isoflavone) pre-treatment increased that activity [49]. A similar observation was reported elsewhere [50]. This difference of results gives rise to the question about the dose-dependent or time-dependent effects of Aβ. Even if we did not deal with this question entirely here, the acute dose of Aβ microinjected (10 µg/side) could be associated with acute neuroinflammation or oxidative stress that promoted a high level of SOD leading to mitigation of ROS generation, as reported also by Suganthy et al. [36]. However, because AD pathogenesis is complex and not fully understood, more studies are needed.

## 5 Conclusion

Our experiment used the hippocampal injection of Aβ_1-40_ to induce an AD-like rat’s model. The neuroprotection actions of the *Chrysophyllum perpulchrum* extract against memory and cognitive deficits and oxidative stress status are thought to be exclusively due to bioactive compounds catechin and two dimer procyanidins (catechin + hexose). However, it is worthwhile to pursue additional pharmacological approaches of *Chrysophyllum perpulchrum* that targets amyloidogenesis and Tau protein hyperphosphorylation, as well as consecutive neuronal signalling pathways involved in AD occurrence. Also, some transgenic or *in vitro* model studies of AD are needed subsequently. The current study is a contribution in the search of candidate therapeutic drugs from African’s traditional pharmacopeia against AD.
